# Double Outlet Right Ventricle: A Rare Finding in a 15‐Month‐Old Female With Failure to Thrive

**DOI:** 10.1002/ccr3.70076

**Published:** 2025-01-06

**Authors:** Bernadette Pedun, Ivaan Pitua, Felix Bongomin, Samuel Bugeza, Rita Nassanga Luzinda

**Affiliations:** ^1^ Uganda Cancer Institute Kampala Uganda; ^2^ Department of Radiology and Radiotherapy, School of Medicine, College of Health Sciences Makerere University Kampala Uganda; ^3^ School of Medicine, College of Health Sciences Makerere University Kampala Uganda; ^4^ Department of Internal Medicine Gulu Regional Referral Hospital Gulu Uganda; ^5^ Department of Medical Microbiology and Immunology Gulu University Gulu Uganda

**Keywords:** cardiac computed tomography, case report, congenital heart defect, double outlet right ventricle, echocardiography

## Abstract

Double outlet right ventricle (DORV) is a rare congenital heart defect where both the aorta and pulmonary artery originate from the right ventricle, often accompanied by additional cardiac anomalies to mitigate circulatory imbalance, though such compensations usually fail. We report a 15‐month‐old infant with recurrent respiratory infections and poor weight gain, referred for computed tomography angiography. Physical examination showed a small, non‐syndromic infant with pallor, tachypnea, irritability, and finger clubbing. Initial imaging revealed cardiomegaly and lung infiltrates; echocardiography and computed tomography angiography confirmed additional intracardiac defects of double superior vena cavae, a hypoplastic aortic arch, hypertrophic right ventricular wall, and a patent ductus arteriosus. Corrective surgery was delayed due to respiratory complications. This case emphasizes the critical need to consider cardiac pathology in pediatric patients with recurrent respiratory symptoms, as untreated DORV can lead to high mortality.

**Summary**

- This case of a 15‐month‐old with double outlet right ventricle (DORV) illustrates the importance of cardiac evaluation in infants with recurrent respiratory symptoms.
- Early detection and multidisciplinary care are essential, as untreated DORV can have severe complications.
- Imaging helps guide management, emphasizing the need for vigilance in pediatric respiratory cases.

## 1 Introduction

Double outlet right ventricle (DORV) is a rare congenital heart defect where both the aorta and pulmonary artery abnormally arise from the right ventricle [1, 2]. This complex anomaly often coexists with other intracardiac defects and is sometimes associated with genetic syndromes such as Trisomy 13 and Trisomy 18 [3]. DORV disrupts normal cardiac structure and blood flow, posing significant physiological challenges.

Although the exact cause of DORV remains unclear, contributing factors may include impaired cardiac development during fetal life, genetic mutations, and exposure to teratogens like alcohol and certain medications [2, 3]. With an incidence of 1%–3% among congenital heart diseases, DORV has a high mortality rate, with over 75% of affected infants not surviving beyond 2 years [1].

DORV is often diagnosed in infancy, with symptoms including respiratory distress, cyanosis, and failure to thrive, though it may occasionally go undetected until later childhood [4]. Early diagnosis typically involves echocardiography to assess structural defects, followed by advanced imaging, such as cardiac MRI or CT, to guide management [5, 6]. Definitive treatment requires staged surgical intervention [7, 8].

In this piece, we delve into the details of this case shedding light on the significance of recognizing DORV early in its presentation contributing to improved management strategies for DORV and better overall outcomes for affected infants.

### 1.1 Case History/Examination

A 15‐month‐old female was referred to radiology department for a computed tomography angiography (CTA) by the pediatric cardiology department due to frequent respiratory infections, including cough, difficulty breathing and feeding, and inadequate weight gain noted since birth. The family history was unremarkable for congenital heart diseases or other significant medical conditions. The patient was born full‐term via an uneventful pregnancy and delivery. There were no reported anomalies on prenatal ultrasounds. Physical examination revealed a non‐syndromic infant with a very small stature with her weight and height below the 5th percentile for her age. The infant appeared pale with tachypnea, irritability and clubbing of the fingers was noted.

**FIGURE 1:**
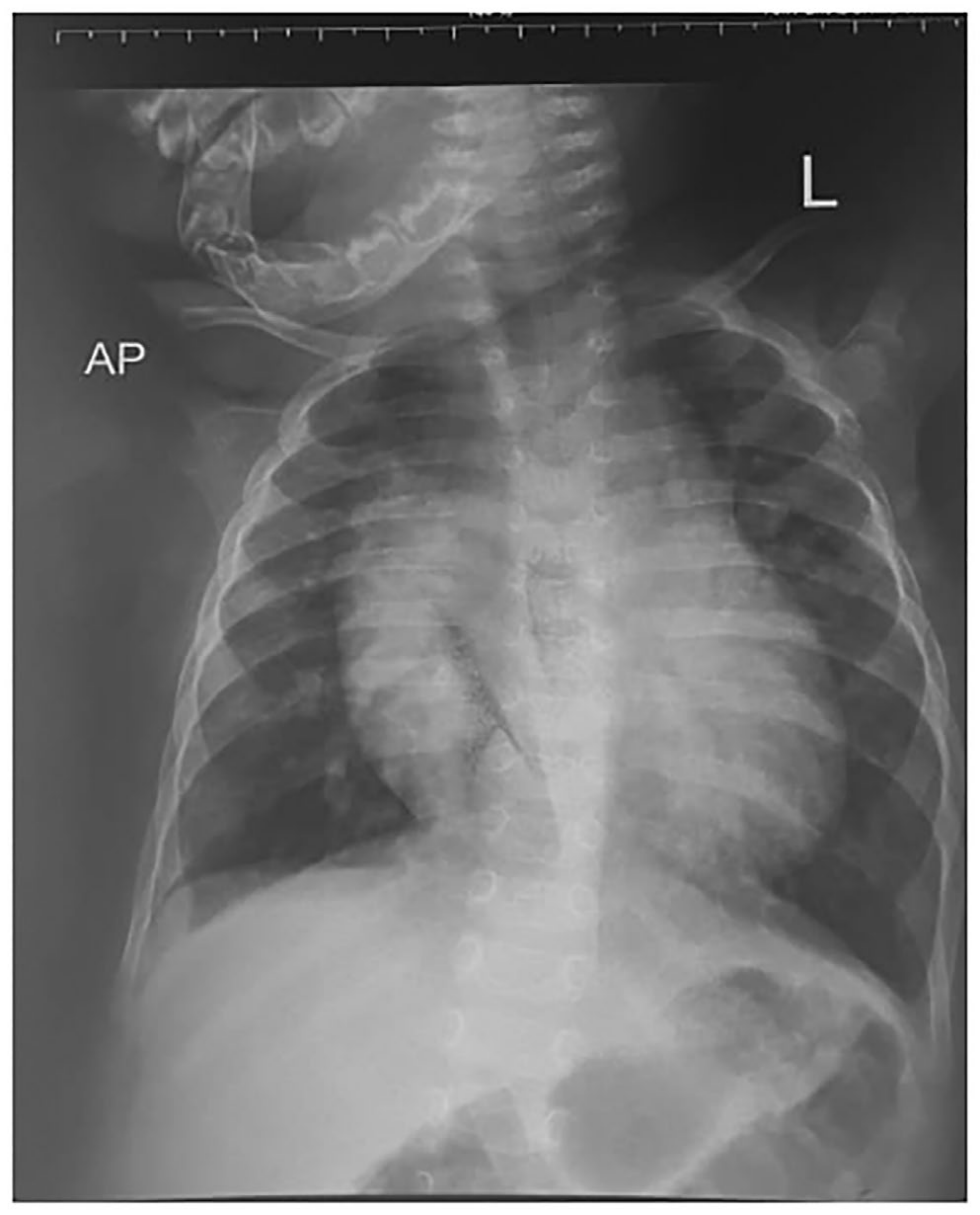
An AP frontal radiograph showing an enlarged cardiac shadow, cardiothoracic ration of 0.7 and widened superior mediastinum, patchy rounded opacities in the para‐cardiac area bilaterally. The rest of the bony ribcage as normal, no defects seen.

An initial chest radiograph was requested by the primary pediatrician and revealed significant cardiomegaly with patchy lung infiltrates bilaterally (Figure 1). Echocardiography was subsequently performed, identifying a DORV with both the aorta and pulmonary artery originating from the right ventricle, alongside a large ventricular septal defect (VSD).

**FIGURE 2:**
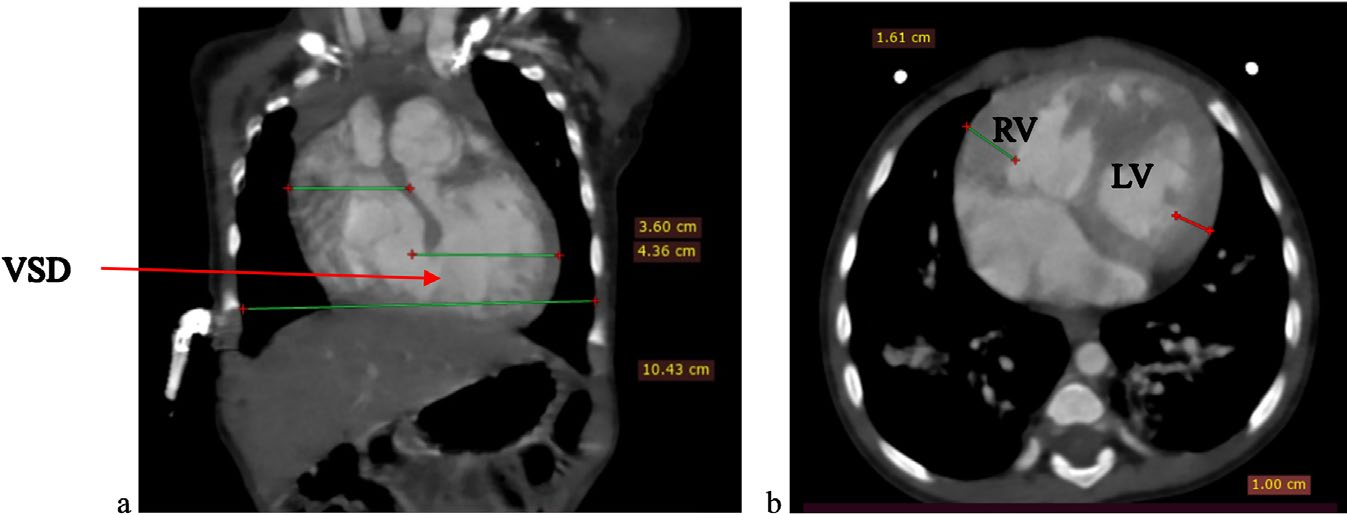
(a, b) shows computed tomography angiography images coronal reformat and axial slices respectively showing the globally enlarged heart with cardiothoracic ratio of 0.76, with a Ventral Septal Defect (VSD) noted in the coronal image, as well as the increased thickness of the right ventricular wall of 1.61 cm in the axial image. LV, left ventricle; RV, right ventricle.

**FIGURE 3:**
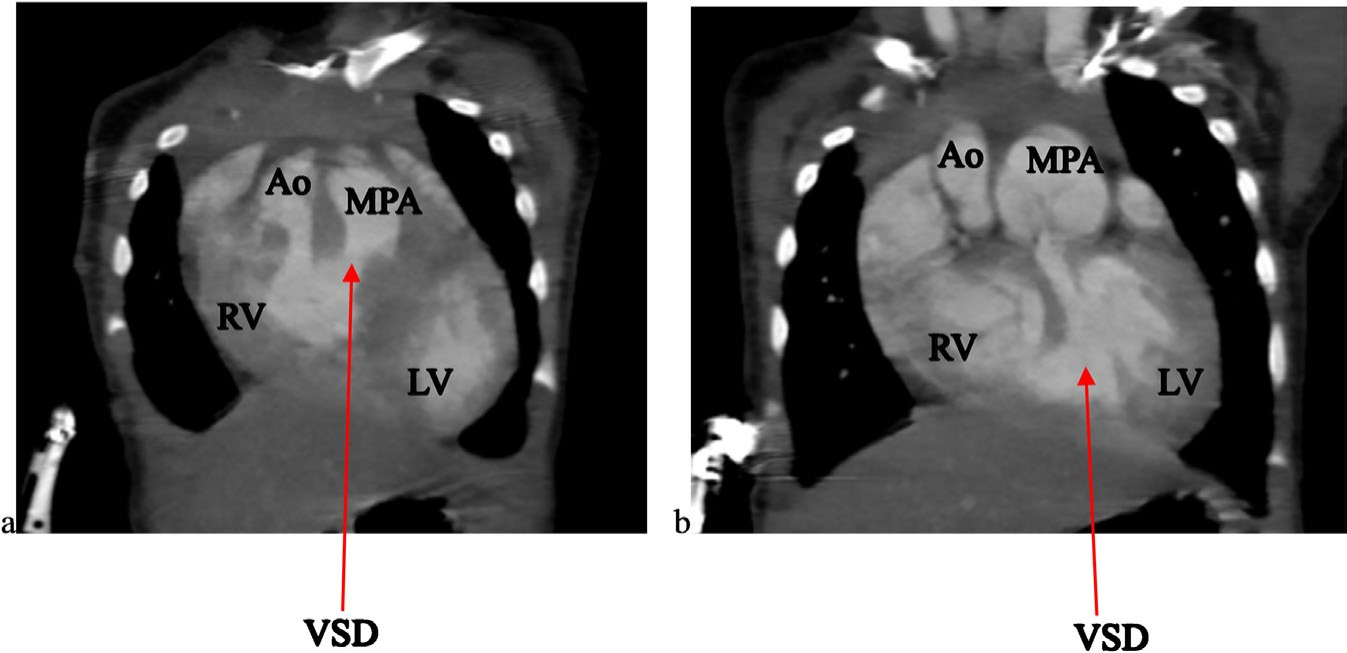
(a, b) shows chest computed tomography angiography coronal reformats at different sections showing the aorta (Ao) arising entirely from the right ventricle (RV), the main pulmonary trunk (MPA) arising from both the right ventricle and the left ventricle (LV) due to a large mid‐septum ventricular septal defect (VSD) which measured 9.49 mm giving the double outlet right ventricle diagnosis. We also see that the MPA is twice the size of the aorta with the MPA: Ao of 2.4:1.

**FIGURE 4:**
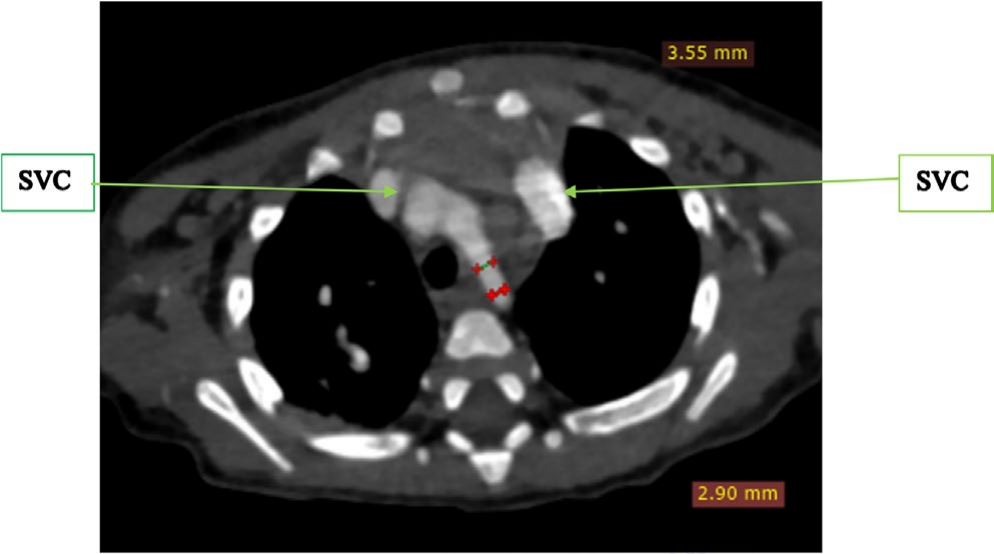
Computed tomography angiography axial slice at the level of the aortic arch showing severely hypoplastic mid and distal arch as compared to the ascending and descending thoracic aorta that measured 1 cm. There is also a left sided superior vena cava in addition to the normally placed right sided superior vena cava (SVC).

**FIGURE 5:**
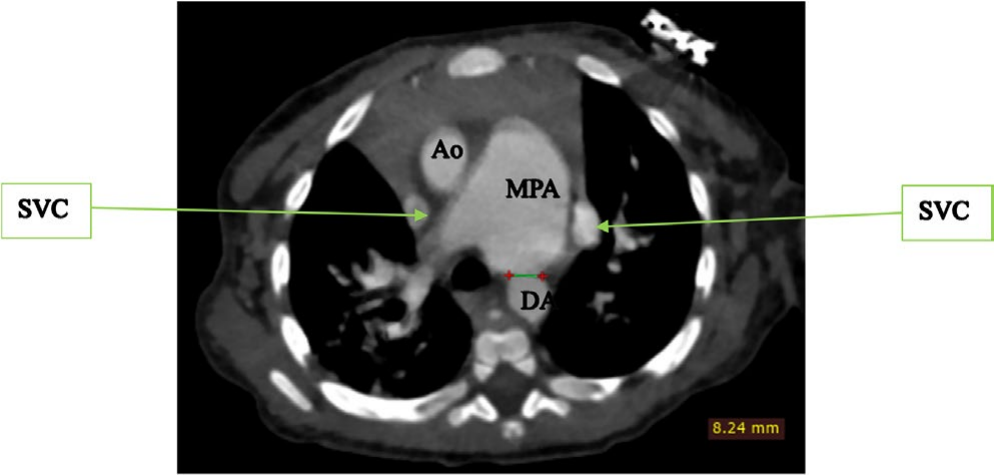
Computed tomography angiography axial slice at the level of the great vessels showing the enlarged main pulmonary trunk (MPA), double superior vena cavae (SVC) and a patent ductus arteriosus between the MPA and the Descending thoracic aorta.

To further evaluate these findings, a cardiac CTA was conducted. The CTA confirmed the DORV and VSD identified on echocardiography, showing a cardiothoracic ratio of 0.76 and hypertrophy of the right ventricular wall measuring 1.61 cm (Figure 2). In addition, the CTA detected anatomic anomalies including double superior vena cavae, a severely hypoplastic mid and distal aortic arch (Figure 4), and an enlarged main pulmonary artery that was approximately twice the diameter of the aorta, leading to an MPA‐to‐Ao ratio of 2.4:1 (Figure 3). A patent ductus arteriosus between the main pulmonary trunk (MPA) and the descending thoracic aorta was also observed (Figure 5). Treatment approach is a multidisciplinary team, including pediatric cardiologists, cardiothoracic surgeons, and pediatric pulmonologists, to formulate the treatment plan however, the patient is yet to have any surgical intervention because of cost implications.

### 1.2 Differential Diagnosis

In this pediatric patient presenting with recurrent respiratory infections, failure to thrive, and signs of cyanosis, the differential diagnosis extended beyond respiratory infections alone and included both cardiac and non‐cardiac causes. Key considerations were other congenital heart defects such as Tetralogy of Fallot, which presents with similar cyanotic features due to right ventricular outflow tract obstruction. VSD and atrioventricular septal defects (AVSD) was also considered, as they can present with respiratory symptoms and cardiomegaly. In cases of failure to thrive with recurrent infections, immunodeficiencies or metabolic disorders are also relevant considerations. However, the presence of specific findings, such as DORV with a large VSD, hypertrophy of the right ventricular wall, and additional vascular anomalies on imaging, strongly supported DORV as the primary diagnosis.

## 2 Conclusion and Results

The case of this 15‐month‐old female with DORV highlights the complexities associated with congenital cardiac anomalies that present with recurrent respiratory infections and failure to thrive. Despite the challenging presentation, imaging findings, including the hypoplastic aortic arch, double superior vena cavae, and patent ductus arteriosus, confirmed the DORV diagnosis, guiding the clinical team in formulating a treatment strategy.

Due to financial limitations, the recommended corrective surgical interventions were not immediately feasible. The patient's management was focused on supportive care to optimize respiratory and nutritional status, stabilizing her for future surgical intervention. The multidisciplinary team, including pediatric cardiology, pulmonology, and cardiothoracic surgery, continued to monitor the patient closely, aiming to perform staged corrective surgery as soon as resources allow. This case reinforces the importance of timely cardiac evaluation in pediatric patients with non‐specific respiratory symptoms and demonstrates that, while immediate intervention may be ideal, interim supportive care and close follow‐up can still provide benefits until definitive treatment is accessible.

## 3 Discussion

DORV is a complex spectrum and rare congenital heart defect where both the aorta and the pulmonary artery arise almost entirely from the right ventricle and greatly mimics several other congenital heart diseases such as tetralogy of Fallot [2, 9]. It results from abnormal cardiac development during the embryonic stage and can manifest in a variety of anatomical and hemodynamic patterns, leading to diverse clinical presentations. The complexity of DORV often involves coexisting anomalies that further complicate its clinical management, as seen in the present case.

There is no known causes or risk factor identified in the case presented here as antenatal and family history were non‐remarkable and no genetic tests have been conducted on her, however existing literature postulates that DORV may result from a myriad of factors such as teratogenic insult, family history of cardiac defects, and sporadic gene mutations that lead to several cardiac defects [2, 4, 9]. The absence of these identifiable risk factors in this case may highlight the sporadic nature of the condition in many instances.

The parents noted that their child had recurrent chest infections and was not gaining weight well as other children about similar age since birth. These findings agree with existing literature regarding the clinical presentation of DORV among other cardiac defects [2, 8]. The case discussed here did not have any syndromic facies identified which differs from findings from a study by [3], who found DORV to be highly associated with syndromes such as Trisomy 13 and 18. The absence of syndromic facies is a notable point, emphasizing the variability of DORV's presentation.

The age of the patient here was 15 months which is not tallying with the mean age at diagnosis for most literature that was 4.08 years, and being female makes her less agreeable to the sex preference of DORV [4]. The clinical features the patient presented with include cyanosis, tachypnea, fatigue, poor feeding, and failure to thrive which is reported also by Bharucha and colleagues [10]. These symptoms, though typical for DORV, can be mistaken for other more common pediatric conditions, which may delay diagnosis in resource‐limited settings.

There is no approved guideline to imaging patients in our setting because the children are initially managed by several lower cadre health professionals whose index of suspicion for cardiac disease in the setting of common respiratory infections is rather low and as such, the incline to request for a plain radiograph of the chest looking out for lung pathology. Following this, the case discussed here was sent for a chest X‐ray which revealed patchy lung infiltrates and an abnormally large cardiac shadow prompting referral to the pediatric cardiologists. The order of events in our case was a deviation from the existing literature which would have been echocardiography then CCTA or CMRI depending on the setting, and the CCTA should be done by a CT machine of at least 128 slices or above [2, 5, 11, 12]. The lack of immediate access to advanced imaging and financial constraints contributed to the unconventional approach.

Echocardiography revealed DORV with a large VSD, while CTA confirmed additional anomalies, including double superior vena cavae, a hypoplastic aortic arch, hypertrophic right ventricular wall, and a patent ductus arteriosus. These findings are consistent with existing literature, which describes the wide range of anatomical anomalies that can accompany DORV [1, 2, 4, 6]. While the CTA provided valuable information, additional imaging, such as MRI, would have further validated these findings, particularly concerning the cardiothoracic ratio and detailed vessel relationships. Unfortunately, due to the patient's financial constraints and subsequent loss to follow‐up, we could not pursue MRI imaging. Although pulmonary hypertension was not directly assessed in this case, the enlarged main pulmonary artery (MPA) seen on CTA (with an MPA‐to‐Ao ratio of 2.4:1) suggests the potential for pulmonary vascular disease. Estimating pulmonary artery pressures via echocardiography could have offered further insights into the hemodynamic impact, but this was not possible due to the patient's unavailability for follow‐up. Further studies focusing on pulmonary hypertension in DORV would add valuable information to the clinical interpretation.

Failure to thrive in this case could have been exacerbated by specific nutritional deficiencies, but such tests were not performed. Despite this, it is well‐documented in literature that failure to thrive in DORV patients is typically influenced by a combination of chronic hypoxia, recurrent infections, and the cardiovascular burden of the defect itself [7, 8]. Nutritional support would be an important part of managing such patients to optimize their growth and development while awaiting corrective surgery. Recurrent respiratory infections are common in DORV patients and can exacerbate the hemodynamic strain caused by the cardiac anomalies. In this case, such infections likely worsened the patient's clinical condition by further impairing oxygenation and increasing the burden on the heart. While a more detailed exploration of the interaction between respiratory infections and cardiac function could have been informative, the loss to follow‐up prevented further assessment.

A clear follow‐up plan is crucial for patients like the one discussed here, where immediate surgical intervention was delayed due to financial constraints. Initially, the patient would receive supportive care to optimize respiratory function, manage recurrent infections, and improve nutritional status, with close monitoring in a hospital setting to assess growth, weight gain, and respiratory health [2, 4, 9]. Before the planned surgery, thorough pre‐operative assessments, including updated imaging (echocardiography, CTA, or MRI), would be necessary to monitor for changes in the patient's condition, such as pulmonary hypertension or worsening heart failure, ensuring the patient is stable for surgery [5, 11]. The first phase of surgical correction would address major anatomical issues, such as the correction of the great vessels (aortic translocation) and closure of the VSD, managed by a multidisciplinary team, with additional procedures, such as repairing the hypoplastic aortic arch and correcting the patent ductus arteriosus, performed as needed [8, 12, 13]. Post‐surgical follow‐up is critical, as DORV patients are at risk for complications like residual cardiac defects, arrhythmias, and pulmonary hypertension, requiring regular cardiac evaluations, including echocardiography, MRI, or CTA, to assess heart function and vessel growth [14]. Pulmonary function would also be monitored for early signs of pulmonary hypertension [14]. Depending on the outcome of the initial surgery and the patient's progress, further interventions may be required in later stages of childhood or adolescence to address any residual defects or complications [12, 13]. This staged approach to surgery, followed by lifelong follow‐up, would provide the best opportunity for long‐term success, improving the patient's quality of life and reducing the risk of mortality. Early detection, timely intervention, and regular monitoring remain the cornerstones of managing DORV in pediatric patients.

This case report was limited by the inability to trace the patient for follow‐up, preventing further hemodynamic assessments such as Doppler flow measurements and pulmonary artery pressure estimations. Financial and resource constraints also prevented additional imaging, such as MRI, and blood tests to assess nutritional deficiencies at the time of presentation. Furthermore, the findings are based on a single case and may not be generalizable to all pediatric DORV patients. Larger studies with more comprehensive diagnostic evaluations are needed for broader conclusions.

Nonetheless, this case emphasizes the importance of early recognition of DORV in pediatric patients presenting with nonspecific respiratory symptoms. Timely diagnosis and intervention, although challenging in resource‐limited settings, are essential for improving outcomes. This case also highlights the need for a multidisciplinary approach, including cardiac surgery, pulmonary care, and nutritional support, to manage the complexities of DORV and its associated anomalies.

## Author Contributions

**Bernadette Pedun:** conceptualization, data curation, formal analysis, investigation, methodology, project administration, resources, software, supervision, validation, visualization, writing – original draft, writing – review and editing. **Ivaan Pitua:** formal analysis, methodology, validation, writing – original draft, writing – review and editing. **Felix Bongomin:** methodology, supervision, validation, writing – original draft, writing – review and editing. **Samuel Bugeza:** formal analysis, investigation, methodology, writing – original draft, writing – review and editing. **Rita Nassanga Luzinda:** data curation, formal analysis, investigation.

## Ethics Statement

No institutional approval was required to publish the case details. The patient's parent provided an informed written consent to participate in the study.

## Consent

Written informed consent for publication of this case report and accompanying images was obtained from the patient's parent.

## Conflicts of Interest

The authors declare no conflicts of interest.

## Data Availability

All relevant data and materials are availed within the text.
